# ﻿Karyotype and genome size variation in white-flowered *Eranthis* sect. *Shibateranthis* (Ranunculaceae)

**DOI:** 10.3897/phytokeys.187.75715

**Published:** 2021-12-31

**Authors:** Elizaveta Yu. Mitrenina, Andrey S. Erst, Lorenzo Peruzzi, Mikhail V. Skaptsov, Hiroshi Ikeda, Vyacheslav Yu. Nikulin, Wei Wang

**Affiliations:** 1 Laboratory of Herbarium, National Research Tomsk State University, Tomsk, Russia National Research Tomsk State University Tomsk Russia; 2 Laboratory of Herbarium, Central Siberian Botanical Garden, Siberian Branch of the Russian Academy of Sciences, Novosibirsk, Russia Laboratory of Herbarium, Central Siberian Botanical Garden, Siberian Branch of the Russian Academy of Sciences Novosibirsk Russia; 3 Department of Biology, Botany Unit, University of Pisa, Pisa, Italy University of Pisa Pisa Italy; 4 South-Siberian Botanical Garden, Altai State University, Barnaul, Russia Altai State University Barnaul Russia; 5 The University Museum, The University of Tokyo, Tokyo, Japan The University of Tokyo Tokyo Japan; 6 Federal Scientific Center of the East Asia Terrestrial Biodiversity, Far Eastern Branch of the Russian Academy of Sciences, Vladivostok, Russia Federal Scientific Center of the East Asia Terrestrial Biodiversity, Far Eastern Branch of the Russian Academy of Sciences Vladivostok Russia; 7 State Key Laboratory of Systematic and Evolutionary Botany, Institute of Botany of the Chinese Academy of Sciences, Beijing, China State Key Laboratory of Systematic and Evolutionary Botany, Institute of Botany of the Chinese Academy of Sciences Beijing China

**Keywords:** Asia, chromosomes, *Eranthis*, genome size, karyotype, Ranunculaceae

## Abstract

Comparative karyomorphological analyses of six out of the eight white-flowered species of Eranthissect.Shibateranthis have been carried out. All studied specimens of *E.byunsanensis*, *E.lobulata*, *E.pinnatifida*, and *E.stellata* had a somatic chromosome number 2*n* = 16 with basic chromosome number *x* = 8. On the contrary, *E.tanhoensis* and *E.sibirica* had a basic chromosome number *x* = 7. The specimens of *E.tanhoensis* were diploid with 2*n* = 14, while the specimens of *E.sibirica* were polyploid with 2*n* = 42. Monoploid chromosome sets of the investigated diploid species had 4–5 metacentric chromosomes and 2–4 submetacentric/subtelocentric/acrocentric chromosomes. The highest level of interchromosomal asymmetry, estimated via CV_CL_, was found in *E.byunsanensis* and *E.pinnatifida*. The highest levels of intrachromosomal asymmetry (M_CA_) and heterogeneity in centromere position (CV_CI_) were found in *E.lobulata* and *E.byunsanensis*, while *E.sibirica* had the most symmetric karyotype. A multivariate PCoA analysis of basic karyotype parameters (2*n*, *x*, THL, CV_CL_, M_CA_, and CV_CI_) highlighted no overlap among species accessions, which was also confirmed by LDA. The average absolute monoploid DNA content (1Cx) of the 23 investigated samples of six *Eranthis* species varied from 9.26 ± 0.25 pg in *E.sibirica* to 15.93 ± 0.32 pg in *E.stellata*. Overall karyological affinity was highlighted between *E.lobulata* and *E.stellata*, on one side, and between *E.byunsanensis* and *E.pinnatifida*, on the other side. Interestingly, there was no significant correlation between total haploid (monoploid) chromosome length (THL) and 1C*x* values in these species.

## ﻿Introduction

Chromosomal analysis is widely used in systematic and evolutionary studies of plants (Yuan and Yang 2006; Guerra 2012; Ilnicki 2014; Baltisberger and Hörandl 2016; Peruzzi et al. 2017). The main features of a karyotype are chromosome number, size and morphology of chromosomes (Astuti et al. 2017). Differences and similarities in karyotypes between taxa may reflect their evolutionary relationship (Shubert 2007; Peruzzi et al. 2009; Escudero et al. 2014; Baltisberger and Hörandl 2016). At present, it is appropriate to use the comparative analysis of karyotypes as part of an integrative approach to solving the issues of systematics and phylogeny (Astuti et al. 2017; Mráz et al. 2019; Erst et al. 2020b).

The genus *Eranthis* Salisb. belongs to Ranunculaceae Juss. tribe Cimicifugeae Torr. & A.Grey (Wang et al. 2009). This genus consists of ten to thirteen early flowering herbaceous perennial species distributed across Southern Europe, Western, Central and temperate Asia (Stefanoff 1963; Rukšāns and Zetterlund 2018; Park et al. 2019; Erst et al. 2020b). This genus generally exhibits a high level of endemism and it is distributed in both mainland and islands. *Eranthis* species seldom co-occur and the size of their distribution range usually varies significantly (Oh and Oh 2019). *Eranthis* is divided into two sections: E.sect.Eranthis and E.sect.Shibateranthis (Nakai) Tamura (Tamura 1987). The species belonging to the first section exhibit perennial tubers or tuberous rhizomes, yellow to orange sepals and yellow petals without pseudonectaries, whereas species of the second section have perennial tubers, white or slightly pink sepals and white petals with pseudonectaries (Tamura 1995; Zetterlund 2018; Park et al. 2019; Rukšāns and Erst et al. 2020b; Huang et al. 2021). The yellow-flowered E.sect.Eranthis includes five species distributed in Southern Europe (*Eranthisbulgarica* (Stef.) Stef., *E.hyemalis* (L.) Salisb.), Western Asia (*E.cilicica* Scott & Kotschy, *E.iranica* Rukšāns & Zetterl) and Central Asia (*E.longistipitata* Regel). The white-flowered E.sect.Shibateranthis includes eight species distributed in temperate Asia. Two species occur in Siberia (*E.sibirica* DC. and *E.tanhoensis* Erst), two in Tibet (*E.albiflora* Franch. and *E.lobulata* W.T.Wang), two in Korea (*E.byunsanensis* B.Y.Sun and *E.pungdoensis* B.U.Oh), one in Japan (*E.pinnatifida* Maxim.), and one is widespread and grows in China, Korea and the Far East of Russia (*E.stellata* Maxim.) (Oh and Oh 2019; Park et al. 2019; Erst et al. 2020b).

The somatic chromosome number 2*n* = 2*x* = 16 has been reported in *Eranthis* in eight species from both sections: *E.byunsanensis* (Kim et al. 2011), *E.cilicica* (Langlet 1932), *E.hyemalis* (Colasante and Ricci 1974; Tak and Wafai 1996; Gömürgen 1998; Caparelli et al. 2007; Erst et al. 2020a), *E.lobulata* (Erst et al. 2019), *E.longistipitata* (Erst et al. 2019), *E.pinnatifida* (Kurita 1955), *E.sibirica* (Gnutikov et al. 2016, 2017), and *E.stellata* (Yuan and Yang 2006). According to another study, *E.stellata* from Russian Far East would have somatic chromosome number 2*n* = 14 (Starodubtsev 1985), and this number was recently found in *E.tanhoensis* (Erst et al. 2020b). Additionally, polyploid cytotypes have been revealed in the genus *Eranthis*, e. g., triploid *E.hyemalis* with 2*n* = 24 (Colasante and Ricci 1974), tetraploid *E.sibirica* that had 2*n* = 32 (Krogulevich 1976) chromosomes, and the same species was recently found actually hexaploid by Erst et al. (2020b). The karyotype has been analyzed for five species: *E.pinnatifida* (Kurita 1955), *E.hyemalis* (Gömürgen 1998), *E.stellata* (Yuan and Yang 2006), *E.sibirica* and *E.tanhoensis* (Erst et al. 2020b).

**Figure 1.**
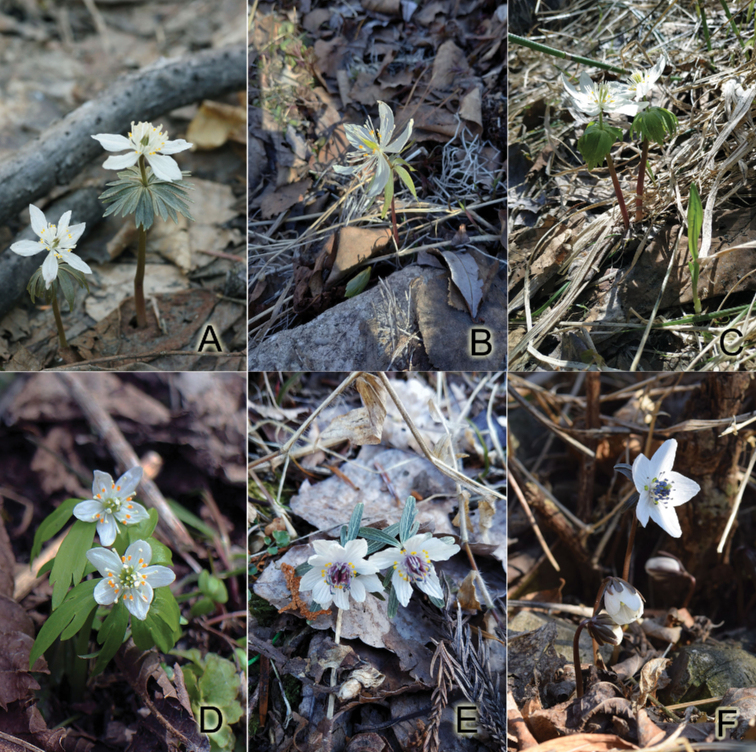
The studied species of white-flowered Eranthissect.Shibateranthis**A***E.stellata* (photo by V.V. Yakubov) **B***E.sibirica* (photo by A.S. Erst); **C***E.tanhoensis* (photo by A.S. Erst) **D***E.lobulata* (photo by K.-L. Xiang) **E***E.pinnatifida* (photo by A.S. Erst) **F***E.byunsanensis* (photo by H.J. Choi).

The genome size (absolute nuclear DNA content), estimated by flow cytometry, is an essential genome feature together with the chromosome number and karyomorphological parameters (Doležel and Bartoš 2005). Flow cytometry can be considered a quick and useful method for understanding taxonomic relationships (Mabuchi et al. 2005; Zonneveld 2010). However, the Plants DNA C-value DataBase (https://cvalues.science.kew.org) contains data on *E.cilicica*, *E.hyemalis*, and *E.pinnatifida* only. This study reports data on comparative karyotype analysis and genome size of six out of eight white-flowered species of Eranthissect.Shibateranthis (Fig. 1): *E.byunsanensis*, *E.lobulata*, *E.pinnatifida*, *E.sibirica*, *E.stellata*, and *E.tanhoensis*.

## ﻿Methods

### ﻿Plant samples

Plant material (tubers) of *E.byunsanensis*, *E.lobulata*, *E.pinnatifida*, *E.sibirica*, *E.stellata*, and *E.tanhoensis* was collected during field investigations in Russia, China, Japan and South Korea during 2018–2020. The list of the samples examined is presented in Table 1. Herbarium specimens were deposited in the E and NS herbaria (herbarium acronyms according to Thiers 2019, continuously updated).

**Table 1.** Chromosome number, ploidy and genome size in white-flowered Eranthissect.Shibateranthis.

N°	Species	Voucher information	2*n*	Ploidy level	1C*x* ± SD (pg)
1	*E.lobulata*	China, Sichuan Province, Jiuding Shan Mountain, 31°32'36.0"N, 103°51'12.0"E, 14 May 2018, L. Zhang	16	2*x*	13.87 ± 0.29
2	E.stellata*	Russia, Primorsky Krai, Vladivostok City, Akademicheskaya Station, 43°11'25.9"N 131°55'31.7"E, 12 Apr 2018, V.V. Yakubov	16	2*x*	15.88 ± 0.31
3	E.stellata*	Russia, Primorsky Krai, Vladivostok City, Malaya Sedanka River, 43°12'36"N, 131°59'24"E, 16 Apr 2019, V.Yu. Nikulin & A.Yu. Nikulin	16	2*x*	15.94 ± 0.34
4	*E.stellata*	Russia, Primorsky Krai, Vladivostok City, forest in the vicinity of "13^th^ km" railway station, 43°11'32"N, 131°55'49"E, 11 Apr 2019, V.Yu. Nikulin & A.Yu. Nikulin	16	2*x*	15.97 ± 0.31
5	E.stellata*	Russia, Primorsky Krai, Vladivostok City, Russkiy Island, 42°59'05.0"N 131°51'51.5"E, 14 May 2019, V.Yu. Nikulin & A.Yu. Nikulin	16	2*x*	14.23 ± 0.23
6	*E.stellata*	China, Jilin Province, Fusong County, Baishan City, Changbai Mt., 852 m alt., 42°06'55.5"N, 127°30'29.0"E, 29 Apr 2019, K. Xiang	16	2*x*	15.99 ± 0.91
7	E.tanhoensis*	Russia, Republic of Buryatiya, Kabansky Raion, Bolshoi Mamai River, mixed forest, 51°23'30.1"N, 104°52'00.8"E, 20 Jun 2019, A.S. Erst, E.Yu. Mitrenina, D.A. Krivenko & O.A. Chernysheva	14	2*x*	12.44 ± 0.27
8	E.tanhoensis*	Russia, Republic of Buryatia, Dulikha River, 51°32'04.9"N, 105°01'43.2"E, 1 May 2019, A.S. Erst, D.A. Krivenko, O.A. Chernysheva	14	2*x*	12.49 ± 0.22
9	E.tanhoensis*	Russia, Buryatia Republic, Kabansky Raion, Tolbazikha River, 51°26'21.06"N, 104°41'09.82"E, 2 May 2019, A.S. Erst, D.A. Krivenko, O.A. Chernysheva	14	2*x*	12.38 ± 0.26
10	E.tanhoensis*	Russia, Irkutsk Oblast, Slyudyansky Raion, Malye Mangaly River, 51°26'48.17"N, 104°34'16.62"E, 02 May 2019, A.S. Erst, D.A. Krivenko, O.A. Chernysheva	14	2*x*	12.07 ± 0.06
11	E.tanhoensis*	Russia, Irkutsk Oblast, Slyudyansky Raion, Semirechka River, 51°28'56.92"N, 104°19'43.47"E, 02 May 2019, A.S. Erst, D.A. Krivenko, O.A. Chernysheva	14	2*x*	12.41 ± 0.29
12	E.tanhoensis*	Russia, Buryatia Republic, Kabansky Raion, Osinovka River (Tankhoi Village), 51°33'06.2"N, 105°05'34.7"E, 01 May 2019, A.S. Erst, D.A. Krivenko, O.A. Chernysheva	14	2*x*	12.56 ± 0.16
13	E.tanhoensis*	Russia, Buryatia Republic, Kabansky Raion, Mishikha River, 51°37'32.6"N, 105°32'03.4"E, 01 May 2019, A.S. Erst, D.A. Krivenko, O.A. Chernysheva	14	2*x*	12.07 ± 0.07
14	E.tanhoensis*	Russia, Buryatia Republic, Kabansky Raion, Shestipalikha River, 51°32'46.4"N, 105°04'28.9"E, 01 May 2019, A.S. Erst, D.A. Krivenko, O.A. Chernysheva	14	2*x*	12.77 ± 0.09
15	E.sibirica*	Russia, Irkutskaya Oblast', Slyudyanksky Raion, vicinity of Slyudyanka Town, mixed forest, 51°38'02.94"N, 103°41'13.90"E, 531 m alt., 02 May 2019, A.S. Erst, D.A. Krivenko & O.A. Chernysheva	42	6*x*	9.23 ± 0.14
16	E.sibirica*	Irkutskaya Oblast', Slyudyanksky Raion, Burovschina River, 51°37'06.00"N, 103°49'16.17"E, 475 m, 20 Jun 2019, A.S. Erst, D.A. Krivenko, E.Yu. Mitrenina & O.A. Chernysheva	42	6*x*	9.27 ± 0.23
17	E.sibirica*	Irkutskaya Oblast', Slyudyanksky Raion, Utulik River, 51°32'50"N, 104°02'45"E, 464 m alt., 20 Jun 2019, A.S. Erst, D.A. Krivenko, E.Yu. Mitrenina & O.A. Chernysheva	42	6*x*	9.22 ± 0.25
18	*E.byunsanensis*	South Korea, Gyeonggi-do, Anyang-si, Suli-san, 37°21'42.8"N, 126°54'01.9"E, 190 m alt., 24 Mar 2019, H. Ikeda, H.-T. Im, K.-S. Chung, M. Fujii, M. Sakamoto & C. Hasekura, N°19032401	16	2*x*	10.75 ± 0.26
19	*E.pinnatifida*	Japan, Saitama Prefecture, Chichibu-shi, Shiroku, near village, 35°57'24"N, 138°59'16"E, 340 m alt., 01 Apr 2019, A.S. Erst, T.V. Erst, H. Ikeda et al., N° 1	16	2*x*	9.87 ± 0.29
20	*E.pinnatifida*	Japan, Mie Prefecture, Inabe-shi, Fujiwara-cho, Ogaito, forest, 35°10'11"N, 136°28'35"E, 180 m alt., 03 Apr 2019, A.S. Erst, T.V. Erst, H. Ikeda et al., N° 2	16	2*x*	9.80 ± 0.46
21	*E.pinnatifida*	Japan, Mie Prefecture, Inabe-shi, Hokusei-cho, Betsumyo, 35°8'23"N, 136°28'20"E, 640 m alt., 04 Apr 2019, A.S. Erst, T.V. Erst, H. Ikeda et al., N° 5	16	2*x*	9.81 ± 0.10
22	*E.pinnatifida*	Japan, Nagano Prefecture, Shiojiri-shi, Hideshio, near station, 36°2'58"N, 137°53'45"E, 825 m alt., 04 Apr 2019, A.S. Erst, T.V. Erst, H. Ikeda et al., N° 6	16	2*x*	9.80 ± 0.43
23	*E.pinnatifida*	Japan, Nagano Prefecture, Shiojiri-shi, Motoyama, pine forest, 36°3'40"N, 137°53'50"E, 800 m alt., 04 Apr 2019, A.S. Erst, T.V. Erst, H. Ikeda et al., N° 7	16	2*x*	9.85 ± 0.27

* population already studied by Erst et al. (2020b) concerning chromosome number and genome size.

### ﻿Karyotype analysis

The comparative karyotype analysis was conducted for 22 populations: one of *E.byunsanensis* and *E.lobulata*, four of *E.pinnatifida*, three of *E.sibirica*, five of *E.stellata*, and eight of *E.tanhoensis* (Table 1). Somatic chromosomes of *Eranthis* were studied from root tip cells. Tubers were germinated in wet moss at ~ 15 °C for 2–4 weeks. Newly formed 1–2 cm long roots were excised and pretreated in 0.5% colchicine solution at 15 °C for 3–4 h. Roots were fixed in a mixture of 96% ethanol and glacial acetic acid (3:1). Root tips were stained with 1% aceto-haematoxylin, and the karyotypes were investigated by the squash method (Smirnov 1968). Chromosomes were counted in 30–100 mitotic cells for each population (a more detailed study was conducted for *E.sibirica* and *E.tanhoensis*). Mitotic metaphase chromosome plates were studied using an Axio Star microscope (Carl Zeiss, Munich, Germany) and photographed using an Axio Imager A.1 microscope (Carl Zeiss, Munich, Germany) with AxioVision 4.7 software (Carl Zeiss, Munich, Germany) and AxioCam MRc5 CCD-camera (Carl Zeiss, Munich, Germany) at 1000× magnification in the Laboratory for Ecology, Genetics and Environmental Protection (Ecogene), National Research Tomsk State University (Tomsk, Russia). KaryoType software (Altınordu et al. 2016) was used for karyotyping, and Adobe Photoshop CS5 (Adobe Systems, USA) and Inkscape 0.92 (USA) were used for image editing.

Karyotype formulas were derived, based on measurements of the photographed mitotic metaphase chromosomes. The measurements were performed on 4–12 metaphase plates per population. We used 2–6 metaphase plates per population with the most condensed chromosomes to calculate mean karyomorphological parameters. The degree of chromosome condensation was estimated from the total haploid length of the chromosome set. The symbols used to describe the karyotypes corresponded to those coined by Levan et al. (1964): m = median centromeric chromosome with arm ratio (r) of 1.0–1.7 (metacentric chromosome); sm = submedian centromeric chromosome with arm ratio of 1.7–3.0 (submetacentric chromosome); st = subterminal centromeric chromosome with arm ratio of 3.0–7.0 (subtelocentric chromosome); t = terminal centromeric chromosome with arm ratio of 7.0 and more (acrocentric chromosome); T = chromosome without obvious short arm (telocentric chromosome). Mean values of arm ratio (r), centromeric indices (CI), mean chromosome length (CL), and relative chromosome length (RL) for each chromosome pair and total haploid length (THL) were determined. In addition, we calculated the Coefficient of Variation of Chromosome Length (CV_CL_; Paszko 2006), Coefficient of Variation of Centromeric Index (CV_CI_; Paszko 2006), and Mean Centromeric Asymmetry (M_CA_; Peruzzi and Eroğlu 2013).

To determine the karyological relationships among taxa, we carried out a multivariate PCoA (Principal Coordinate Analysis) using Gower's general coefficient of similarity, including six basic karyomorphological parameters (2*n*, *x*, THL, M_CA_, CV_CL_, and CV_CI_) in the data matrix (Peruzzi and Altınordu 2014), by plotting every single metaphase. Then, we also subjected the same data matrix to LDA (Linear Discriminant Analysis) to test the diagnosability of the six species on karyomorphological grounds. Finally, we tested the Spearman correlation between THL and 1C*x* for each species, using mean data. To perform PCoA, LDA and correlation tests, the software Past 4.06b (Hammer et al. 2001; Hammer 2021), freely available online, was used.

### ﻿Flow cytometry

Flow cytometry with propidium iodide (PI) staining was used to determine the absolute DNA content. In this study, we have determined this parameter in representatives of four *Eranthis* species: *E.byunsanensis*, *E.lobulata*, *E.pinnatifida* and *E.stellata* from 10 different populations (Table 1). Silica-gel-dried leaf material (0.5–1.0 cm^2^) was chopped with a sharp razor blade in a 1 ml cold nuclei extraction buffer composed of 50 mM Hepes, 10 mM sodium metabisulphite, 10 mM MgCl_2_, 0.5% polyvinylpyrrolidone, 0.1% bovine serum albumin, 0.3% Tween 20, 0.2% Triton X-100, 50 μg/ml RNase, 1 μg/ml β-mercaptoethanol and 50 μg/ml propidium iodide (PI). The samples were filtered through 50 μm nylon membranes into sample tubes and incubated in the dark at 4 °C for 15 min. The samples were measured using a Partec CyFlow PA flow cytometer equipped with a green laser at 532 nm wavelength. The absolute nuclear DNA content, the 2C-value according to Greilhuber et al. (2005), was calculated as the ratio of the mean fluorescence intensity of the sample nuclei to that of the external standard multiplied by the total nuclear DNA content of the standard. The possible effect of secondary metabolites on the binding of the intercalating dye was evaluated by measuring the fluorescence of *Alliumfistulosum* L. leaf samples prepared as described above, but with the addition of the supernatant from *Eranthis* samples centrifuged without PI. The samples were measured three times at 10 min intervals. If no variation in the average values of the detection channels was observed for the *A.fistulosum* peak, the effect of secondary metabolites was considered negligible. The 1C*x*-value (monoploid DNA content *sensu*Greilhuber et al. 2005) was calculated by dividing the 2C-value by the ploidy level of the species. The species, used as external standards, were *Zamioculcaszamiifolia* Engl., 2C = 48.35 pg and *Viciafaba* L. "Inovec" 2C = 26.90 pg (Doležel et al. 1992; Skaptsov et al. 2016). We used the Statistica 8.0 software (StatSoft, Inc.), Flowing Software 2.5.1 (Turku Centre for Biotechnology) and CyView software (Partec, GmbH) for data analyses. Flow cytometry was performed at the Laboratory for Bioengineering of the South-Siberian Botanical Garden, Altai State University (Barnaul, Russia).

### ﻿Results

﻿**Karyotypes**

**Figure 2.**
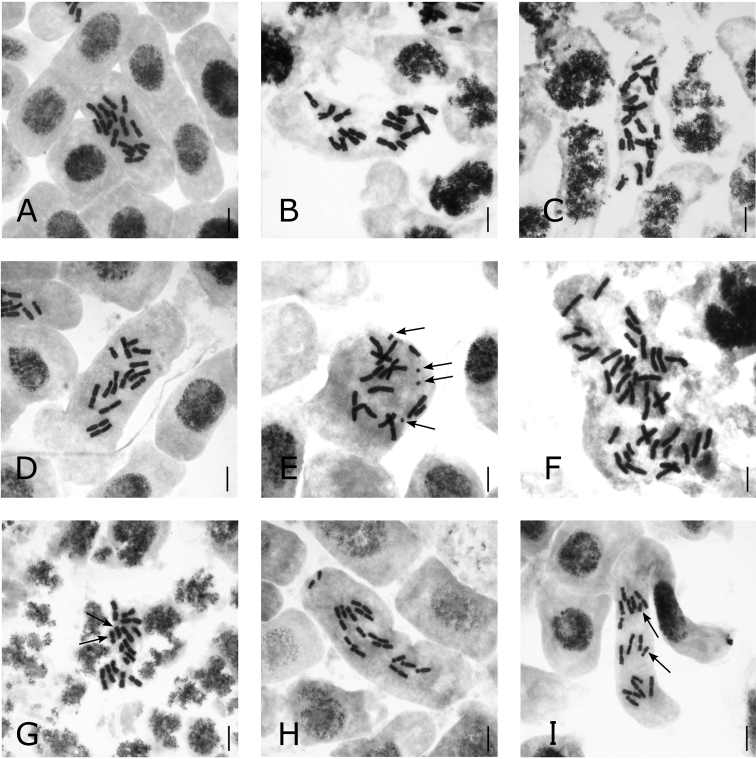
Mitotic metaphase plates of white-flowered Eranthissect.Shibateranthis**A***E.lobulata*, 2*n* = 16 **B***E.stellata* (pop. 2), 2*n* = 16 **C***E.stellata* (pop. 6), 2*n* = 16 **D***E.tanhoensis* (pop. 12), 2*n* = 14 **E***E.tanhoensis* (pop. 10), 2*n* = 14+0–8B (arrows point at **B** chromosomes) **F***E.sibirica* (pop. 15), 2*n* = 42 **G***E.byunsanensis*, 2*n* = 16 (arrows point at the heteromorphic chromosome pair) **H***E.pinnatifida* (pop. 21), 2*n* = 16 **I***E.pinnatifida* (pop. 20), 2*n* = 16 (arrows point at heteromorphic chromosome pair). Scale bars: 10 μm. Microphotographs by E.Yu. Mitrenina.

**Figure 3.**
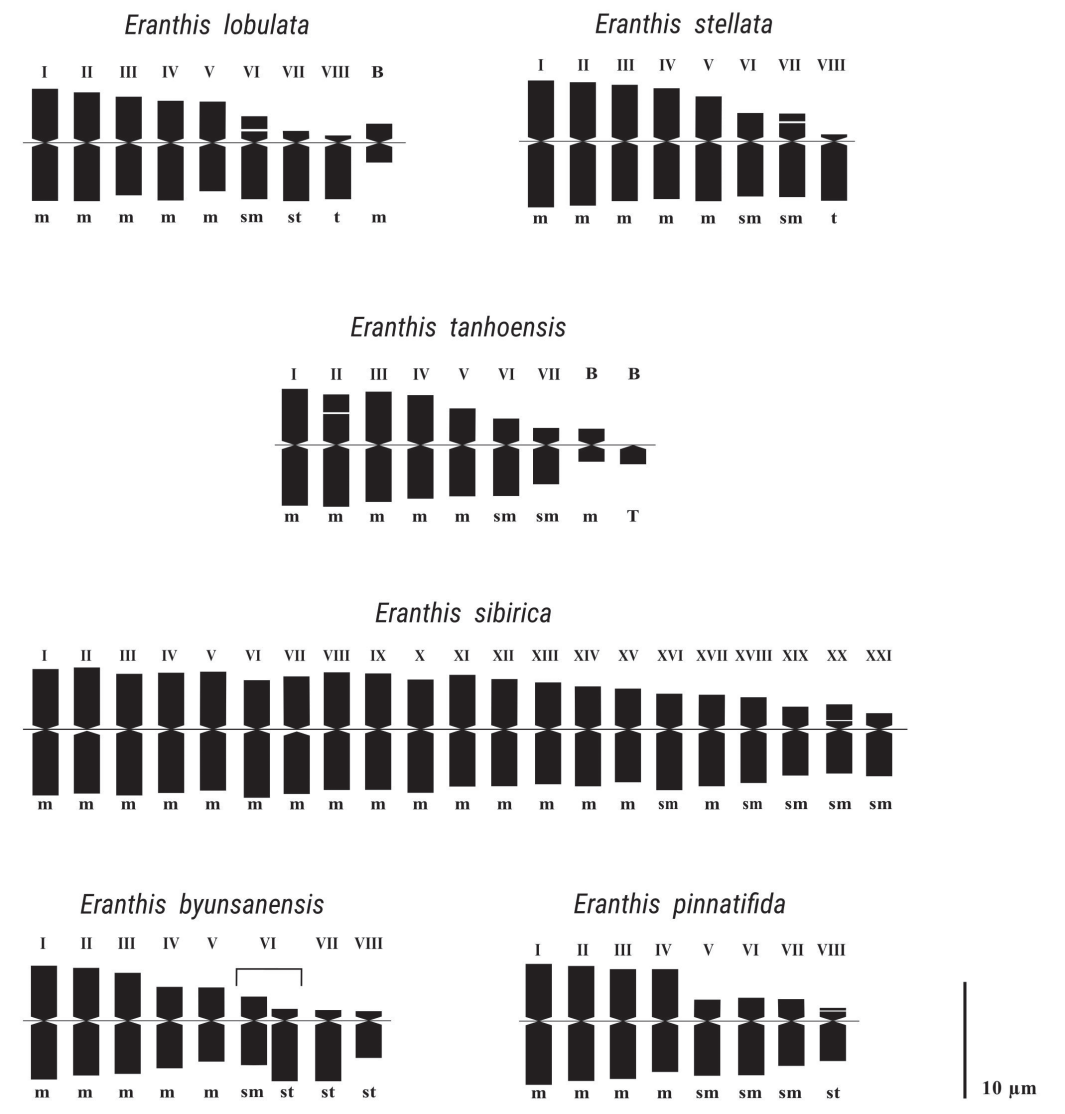
Haploid idiograms of white-flowered Eranthissect.Shibateranthis species. I–VIII – chromosome pairs; m – metacentric chromosome; sm – submetacentric chromosome; st – subtelocentric chromosome; t – acrocentric chromosome; T – telocentric chromosome; B – B chromosome.

Karyomorphometric data, microphotographs of metaphase plates and idiograms for the studied species are presented in Tables 2, 3 and Figs 2, 3.

#### ﻿ Eranthis lobulata

Taxon classificationPlantaeRanunculalesRanunculaceae

92F46FE3-0F93-5811-9905-1CCD40A3021E

##### Notes.

The somatic and basic chromosome numbers in *E.lobulata*, endemic to China, are 2*n* = 16 and *x* = 8, respectively (Table 1; Fig. 2A). Five pairs of chromosomes (I–V) are metacentric, and three pairs (VI–VIII) are submetacentric, subtelocentric and acrocentric (Tables 2 and 3; Fig. 3). A pair of submetacentric chromosomes exhibits a secondary constriction. We also found a single small B chromosome in some cells. These Bs are metacentric, about 2.5 μm long. The karyotype formula of *E.lobulata* is 2*n* = 2*x* = 16 = 10m + 2sm^sat^ + 2st + 2t + 0–1B.

**Table 2.** Karyomorphological parameters in white-flowered Eranthissect.Shibateranthis.

Species	Chromosome pair	CL (µm)	r	CI	RL (%)	Type
***E.lobulata***	I	8.46 ± 0.42	1.07 ± 0.04	0.48	7.80	m
	II	8.19 ± 0.31	1.16 ± 0.09	0.46	7.55	m
	III	7.43 ± 0.30	1.17 ± 0.07	0.46	6.85	m
	IV	7.38 ± 0.16	1.36 ± 0.10	0.42	6.80	m
	V	7.00 ± 0.29	1.28 ± 0.05	0.44	6.45	m
	VI	6.11 ± 0.15	2.05 ± 0.09	0.33	5.63	sm^sat^
	VII	5.05 ± 0.21	5.04 ± 0.51	0.17	4.66	st
	VIII	4.62 ± 0.24	8.35 ± 0.84	0.11	4.26	t
***E.stellata* (pop. 2)**	I	9.61 ± 0.34	1.07 ± 0.04	0.48	7.84	m
	II	9.29 ± 0.31	1.07 ± 0.04	0.48	7.58	m
	III	8.85 ± 0.39	1.06 ± 0.03	0.49	7.22	m
	IV	8.31 ± 0.42	1.06 ± 0.04	0.49	6.78	m
	V	7.89 ± 0.16	1.33 ± 0.07	0.43	6.44	m
	VI	6.21 ± 0.25	2.00 ± 0.19	0.33	5.06	sm
	VII	6.13 ± 0.40	2.14 ± 0.18	0.32	5.00	sm^sat^
	VIII	5.01 ± 0.34	7.86 ± 0.38	0.11	4.08	t
***E.tanhoensis* (pop. 12)**	I	8.68 ± 0.36	1.09 ± 0.05	0.48	8.74	m
	II	8.56 ± 0.41	1.23 ± 0.06	0.45	8.62	m^sat^
	III	8.16 ± 0.29	1.07 ± 0.05	0.48	8.21	m
	IV	7.73 ± 0.35	1.07 ± 0.05	0.48	7.78	m
	V	6.63 ± 0.46	1.37 ± 0.11	0.42	6.67	m
	VI	5.72 ± 0.46	1.92 ± 0.14	0.34	5.76	sm
	VII	4.19 ± 0.38	2.34 ± 0.15	0.30	4.22	sm
***E.sibirica* (pop. 15)**	I	9.51 ± 0.24	1.08 ± 0.04	0.48	2.88	m
	II	9.47 ± 0.29	1.03 ± 0.02	0.49	2.87	m
	III	9.20 ± 0.06	1.17 ± 0.03	0.46	2.78	m
	IV	9.13 ± 0.13	1.10 ± 0.06	0.48	2.76	m
	V	9.00 ± 0.11	1.05 ± 0.02	0.49	2.72	m
	VI	8.91 ± 0.14	1.39 ± 0.12	0.42	2.70	m
	VII	8.88 ± 0.07	1.20 ± 0.03	0.45	2.69	m
	VIII	8.87 ± 0.16	1.05 ± 0.03	0.49	2.68	m
	IX	8.67 ± 0.10	1.08 ± 0.05	0.48	2.62	m
	X	8.47 ± 0.09	1.27 ± 0.09	0.44	2.56	m
	XI	8.44 ± 0.15	1.07 ± 0.03	0.48	2.55	m
	XII	8.14 ± 0.13	1.16 ± 0.02	0.46	2.46	m
	XIII	7.71 ± 0.04	1.18 ± 0.09	0.46	2.33	m
	XIV	7.46 ± 0.15	1.35 ± 0.15	0.43	2.26	m
	XV	7.26 ± 0.21	1.70 ± 0.06	0.37	2.20	sm
	XVI	7.10 ± 0.04	1.28 ± 0.03	0.44	2.15	m
	XVII	6.89 ± 0.05	1.61 ± 0.05	0.38	2.08	m
	XVIII	6.45 ± 0.31	1.70 ± 0.08	0.37	1.95	sm
	XIX	5.36 ± 0.23	1.97 ± 0.09	0.34	1.62	sm
	XX	5.24 ± 0.25	1.74 ± 0.03	0.37	1.59	sm^sat^
	XXI	5.08 ± 0.34	2.29 ± 0.14	0.30	1.54	sm
***E.byunsanensis***	I	8.59 ± 0.19	1.05 ± 0.03	0.49	8.55	m
	II	8.13 ± 0.31	1.06 ± 0.04	0.49	8.09	m
	III	7.65 ± 0.13	1.07 ± 0.04	0.48	7.61	m
	IV	6.18 ± 0.09	1.40 ± 0.05	0.42	6.15	m
	V	5.68 ± 0.21	1.19 ± 0.05	0.46	5.65	m
	VI	5.44 ± 0.19	5.22 ± 0.30	0.16	5.41	st
		5.19 ± 0.08	1.74 ± 0.05	0.37	5.17	sm
	VII	5.20 ± 0.13	5.64 ± 0.19	0.15	5.17	st
	VIII	3.52 ± 0.07	4.06 ± 0.37	0.20	3.50	st
***E.pinnatifida* (pop. 21)**	I	9.24 ± 0.18	1.12 ± 0.02	0.47	8.58	m
	II	8.63 ± 0.24	1.08 ± 0.06	0.48	8.02	m
	III	8.25 ± 0.31	1.13 ± 0.03	0.47	7.66	m
	IV	6.62 ± 0.12	1.37 ± 0.07	0.42	6.15	m
	V	6.24 ± 0.26	2.77 ± 0.14	0.27	5.80	sm
	VI	5.88 ± 0.18	2.38 ± 0.07	0.30	5.46	sm
	VII	5.04 ± 0.11	1.95 ± 0.13	0.34	4.68	sm
	VIII	3.92 ± 0.09	3.10 ± 0.29	0.24	3.64	st^sat^

Notes: CL – chromosome length, mean value ± standard deviation; r – arm ratio, mean value ± standard deviation; CI – centromeric index; RL – relative chromosome length; m – metacentric chromosome; sm – submetacentric chromosome; st – subtelocentric chromosome; t – acrocentric chromosome; ^sat^ – chromosome showing secondary constriction.

#### ﻿ Eranthis stellata

Taxon classificationPlantaeRanunculalesRanunculaceae

6FF7E951-2BD3-5D89-9B8F-053AB8755641

##### Notes.

In all five studied populations of *E.stellata* from Primorsky Krai of Russia and Jilin Province of China, the somatic and basic chromosome numbers are 2*n* = 16 and *x* = 8, respectively (Table 1; Fig. 2B–C). Five pairs of chromosomes (I–V) are metacentric, two pairs (VI–VII) are submetacentric, and one pair (VIII) is acrocentric (Tables 2 and 3; Fig. 3). A pair of submetacentric chromosomes (VII) exhibits a secondary constriction. The karyotype formula of *E.stellata* is 2*n* = 2*x* = 16 = 10m + 2sm + 2sm^sat^ + 2t. No B was observed in this species. Here, we present the results of the karyomorphological analysis of *E.stellata* from the "Academicheskaya Station" population (pop. 2).

**Table 3.** Karyotype parameters in white-flowered Eranthissect.Shibateranthis.

**Species**	**N**	**Ploidy level**	**2*n***	**Karyotype formula**	**THL**	**CV_CL_**	**M_CA_**	**CV_CI_**
*E.lobulata*	6	2*x*	16	10m + 2sm^sat^ + 2 st + 2t + 0–1B	54.24 (0.92)	19.68 (0.36)	28.15 (0.80)	38.05 (1.09)
*E.stellata* (pop. 2)	5	2*x*	16	10m + 2sm + 2sm^sat^ + 2t	61.30 (1.91)	20.77(0.84)	21.63(0.51)	31.69(0.83)
*E.tanhoensis* (pop. 12)	5	2*x*	14	8m + 2m^sat^ + 4sm + 0–8B	49.67 (2.02)	22.11 (1.10)	15.46 (0.76)	16.32 (1.24)
*E.sibirica* (pop. 15)	2	6*x*	42	32m + 8sm + 2sm^sat^	165.24 (0.85)	17.55(0.87)	13.41(0.65)	12.87(0.18)
*E.byunsanensis*	4	2*x*	16	10m + 1sm + 5 st	50.26 (0.83)	25.69 (0.66)	26.43 (0.23)	37.18 (1.11)
*E.pinnatifida* (pop. 21)	5	2*x*	16	8m + 6sm + 2 st^sat^	53.82 (0.81)	25.81 (0.89)	25.16 (0.72)	24.90 (0.88)

Notes: THL – total haploid length, CV_CL_ – Coefficient of Variation of Chromosome Length, M_CA_ – Mean Centromeric Asymmetry, CV_CI_ – Coefficient of Variation of Centromeric Index; mean value (standard deviation). m – metacentric chromosome; sm – submetacentric chromosome; st – subtelocentric chromosome; t – acrocentric chromosome; ^sat^ – satellite chromosome; B – B chromosome.

#### ﻿ Eranthis tanhoensis

Taxon classificationPlantaeRanunculalesRanunculaceae

B3B44D92-FCB5-5C29-AAF2-CEEF5FC4D786

##### Notes.

In all eight studied populations of *E.tanhoensis*, Siberian endemic species, the somatic and basic chromosome numbers are 2*n* = 14 and *x* = 7, respectively (Table 1; Fig. 2D–E). Five pairs of chromosomes (I–V) are metacentric, two pairs (VI–VII) are submetacentric (Tables 2 and 3; Fig. 3). A pair of metacentric chromosomes (II) exhibited a secondary constriction. We found small B chromosomes in plants from two populations (pops 10 and 13). The maximum number of Bs in root tip cells appeared to be 8. They were represented by small metacentric and dot-shaped chromosomes, which are obviously telocentric. The karyotype formula of *E.tanhoensis* is 2*n* = 2*x* = 14 = 8m + 2m^sat^ + 4sm + 0–8B. Here, we present the results of the karyomorphological analysis of *E.tanhoensis* from the "Tanhoi Village" population (pop. 12).

#### ﻿ Eranthis sibirica

Taxon classificationPlantaeRanunculalesRanunculaceae

ED1DFAAE-0112-5A26-BC9B-2FA8F508B44F

##### Notes.

The somatic chromosome number of *E.sibirica*, another endemic species from Siberia, is 2*n* = 42. The chromosome set of the species includes metacentric and submetacentric types of chromosomes. The karyotype formula of *E.sibirica* is 2*n* = 6*x* = 42 = 32m + 8sm + 2sm^sat^. Here, we present the results of the karyomorphological analysis of *E.sibirica* from the "Slyudyanka Town" population (pop. 15) (Tables 2 and 3; Figs 2F and 3).

#### ﻿ Eranthis byunsanensis

Taxon classificationPlantaeRanunculalesRanunculaceae

47C1D658-F296-53F2-BF53-587F95692C7A

##### Notes.

The chromosome set of the Korean endemic *E.byunsanensis* includes five pairs of metacentric chromosomes (I–V), one submetacentric (in the "pair" VI) and five subtelocentric chromosomes (in the "pair" VI and pairs VII–VIII) (Tables 2 and 3; Figs 2G and 3). The karyotype formula of *E.byunsanensis* is 2*n* = 2*x* = 16 = 10m + 1sm + 5st.

#### ﻿ Eranthis pinnatifida

Taxon classificationPlantaeRanunculalesRanunculaceae

8D5615FC-2175-593D-A59A-01633579A0FD

##### Notes.

The Japanese endemic *E.pinnatifida*, unlike other related species, has four rather than five pairs of metacentric chromosomes (I–IV) and four rather than three pairs of submetacentric (V–VII) and subtelocentric chromosomes (VIII). The karyotype formula of the plants from three studied populations (pops 19, 21 and 22) is 2*n* = 2*x* = 16 = 8m + 6sm + 2st^sat^. These plants have secondary constrictions and small satellites at terminal regions of short arms of the pair VIII (Table 2; Figs 2H and 3). Specimens from the fourth population (pop. 20) have a pair of heteromorphic chromosomes (VIII) represented by one metacentric and one subtelocentric chromosome (Fig. 2I). The karyotype formula of these plants is 2*n* = 2x = 16 = 8m + 1m^sat^ + 6sm + 1st^sat^. In these plants, the secondary constriction in the metacentric homologue to the VIII pair is located in the pericentromeric region. Here we present the results of the karyomorphological analysis of *E.pinnatifida* from the "Inabe-shi" population (pop. 21).

### ﻿Karyotype structure

**Figure 4.**
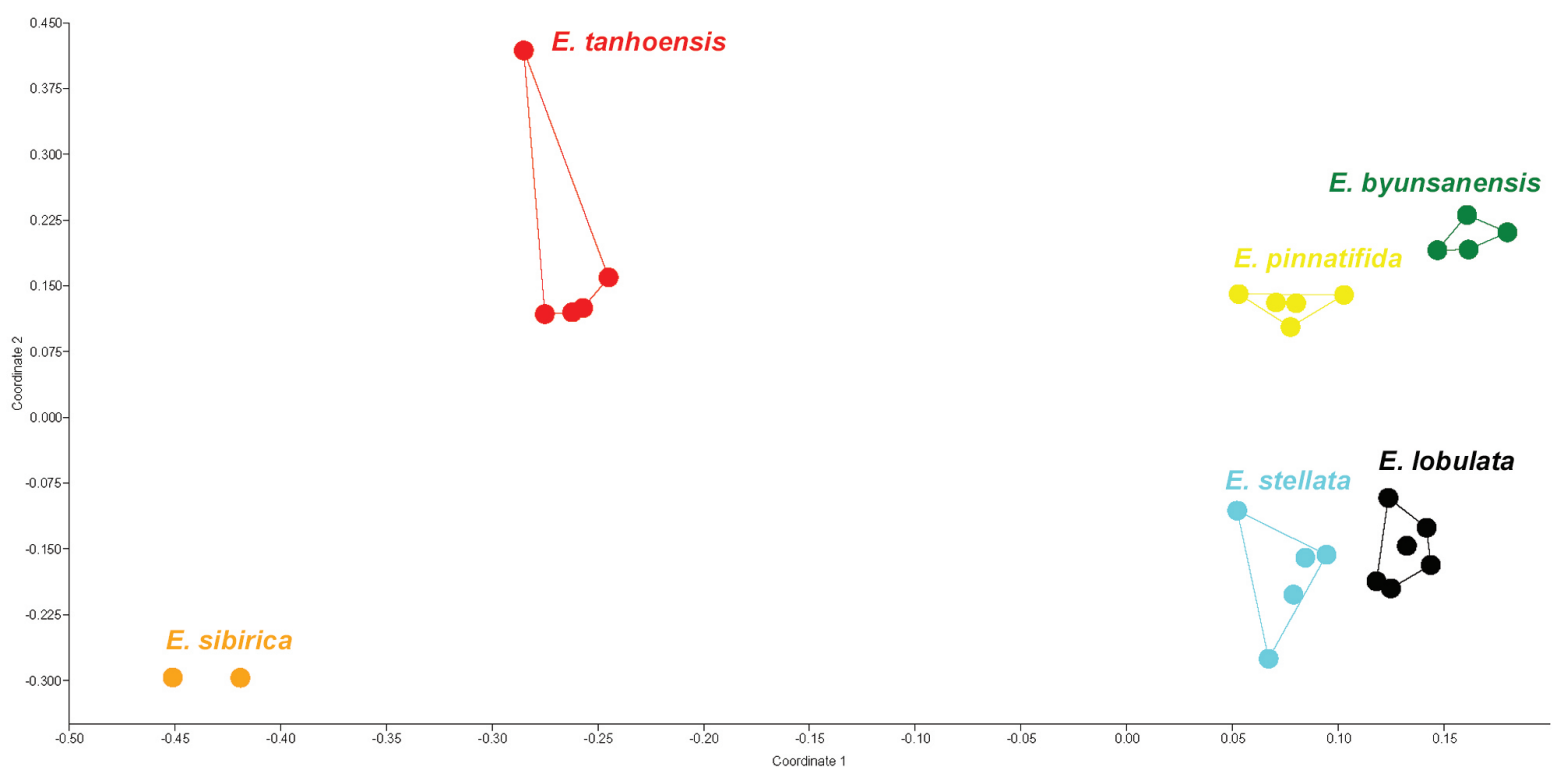
PCoA (Coordinate 1, 65.31% of variance explained vs. Coordinate 2, 16% of variance explained) based on six karyological parameters of white-flowered Eranthissect.Shibateranthis species.

The highest level of interchromosomal asymmetry, estimated via CV_CL_, was found in *E.byunsanensis* and *E.pinnatifida*. The highest levels of intrachromosomal asymmetry (M_CA_) and heterogeneity in centromere position (CV_CL_) were found in *E.lobulata* and *E.byunsanensis*, while *E.sibirica* had the most symmetric karyotype (Table 3). We analyzed 27 accessions (metaphase plates) by PCoA (cumulative variance explained by the first two axes: 81.31%). No overlap among species was evident (Fig. 4). Indeed, LDA correctly attributed objects (accessions) to the six species in 100% of cases (jackknifed).

### ﻿Genome size

The absolute nuclear DNA content for 23 studied populations of six species of *Eranthis* is presented in Table 1. There was no significant correlation (r = 0.51, p = 0.29) between mean 1C*x* values and total haploid (monoploid) chromosome length (THL) in these species. Indeed, for instance, while the 1C*x* value is the smallest in the hexaploid *E.sibirica*, THL in this species is higher than in the diploid *E.tanhoensis*, sharing the same basic chromosome number *x* = 7 (Table 3).

## ﻿Discussion

### ﻿Karyotype structure in *Eranthis*

According to our results and other data (Kurita 1955; Tak and Wafai 1996; Gömürgen 1998; Yuan and Yang 2006), chromosome sets of different species of *Eranthis* share some common features, albeit showing some species-specific peculiarities, which allow a clear-cut distinction among species based on karyo-morphological features according to LDA (see also Fig. 4). The traits of the karyotype within each species are sufficiently stable. However, in some cases, polymorphism was observed in the chromosome morphology, for instance, in *E.pinnatifida*. The karyotypes of *E.byunsanensis* and *E.lobulata* were described here for the first time.

The chromosomes of *Eranthis* belong to the *Ranunculus*-type (Langlet 1932). The karyotypes of *E.lobulata* and *E.stellata* are similar. Both species show a chromosome pair with a very small, not always visible, short arm. In the other four studied species of *Eranthis*, no chromosome of this type was found. The secondary constrictions in *E.lobulata* and *E.stellata* are localized in the short arms of submetacentric chromosome pairs. Different localization of secondary constrictions in these species (Fig. 3) is possibly due to a paracentric inversion. Previously, the karyotype of *E.stellata* from China (Jilin Province) was studied by Yuan and Yang (2006). These authors described its formula as 2*n* = 2*x* = 16 = 10m + 2sm + 2st + 2T. In contrast to our data, they assigned pair VII to subtelocentric rather than submetacentric chromosome type. Our data show that the arm ratio of this chromosome pair is 2.14 ± 0.18 (Table 2), congruent with a sm chromosome-type. They also did not find a short arm in pair VIII and referred it to T-type (telocentric chromosomes). We found short arms in this VIII chromosome pair, which led us to classify it as chromosomes of t-type (acrocentric chromosomes).

Two species, endemic to Siberia, *E.sibirica* and *E.tanhoensis*, show atypical dysploid basic chromosome number for *Eranthis* (*x* = 7) and exhibit hexaploid (2*n* = 42) and diploid (2*n* = 14) cytotypes, respectively (Erst et al. 2020b). Since there are different definitions of the term "basic chromosome number (*x*)" concerning polyploids (Peruzzi 2013), we clarify that, in the study, we mean, "*x*" as "chromosome number found in the gametes of their diploid relatives", according to Darlington (1958). A recent phylogenetic study (Xiang et al. 2021) found that *E.tanhoensis* and *E.sibirica* are closely related species that formed separate groups with basic chromosome number *x* = 7 within the North Asian clade of *Eranthis*. The same basic chromosome number *x* = 7 with 2*n* = 14 was previously reported in the genus *Eranthis* for *E.stellata* (Starodubtsev 1985), albeit this author does not provide any microphotograph of the metaphase plate. We re-analyzed plants from the same area (pop. 3; Russia, Primorsky Krai, Malaya Sedanka River), but we found a somatic chromosome number 2*n* = 16. At the same time, previous studies on *E.sibirica* reported 2*n* = 16 (Gnutikov et al. 2016, 2017) and 2*n* = 32 (Krogulevich 1976) chromosomes. However, the diploid plants described in these studies apparently refer to the recently described species *E.tanhoensis*. Some populations of this species show B chromosomes that researchers may have identified as regular chromosomes. In addition, a pair of metacentric chromosomes show large satellites, which, when using the squash method, are sometimes detached and can be misidentified as small telocentric chromosomes. Based on the large amount of material analyzed and on careful analysis of chromosome morphology, we conclude that the basic chromosome number of the studied populations of *E.tanhoensis* and *E.sibirica* is *x* = 7. However, we do not rule out the possible occurrence of different cytotypes in plants from Siberia.

The karyotypes of the two related species, endemic to Korea and Japan, also show peculiar features. *Eranthisbyunsanensis* has a heteromorphic pair of chromosomes (VI). Unfortunately, we had material from a single population of this species. Therefore, we cannot conclude whether this feature is characteristic of the whole species or just a heterozygous chromosomal mutation. *Eranthispinnatifida* has another feature that distinguishes it from other diploid species: four pairs of isobrachial chromosomes and four pairs of heterobrachial chromosomes. Our results concerning this species are consistent with the data published by Kurita (1955). Among the four *E.pinnatifida* populations studied, one population (pop. 20) shows a heteromorphic pair of chromosomes. In this case, we are sure that this mutation is just a polymorphic variant.

Carta et al. (2020) estimated *x* = 7 as the most likely ancestral basic chromosome number in Ranunculaceae. However, we hypothesize that, in Siberian species, *E.sibirica* and *E.tanhoensis*, the basic chromosome number evolutionarily reduced from *x* = 8 to *x* = 7 and not vice versa. This hypothesis is because most of the tribe Cimicifugeae members (i.e., *Actaea*, *Anemonopsis*, *Beesia*, *Cimicifuga*, *Souliea* and closely related *Helleborus*; Wang et al. 2009) have *x* = 8 (Rice et al. 2015). In addition, it has been established that *Eranthis* originated in East Asia and then dispersed to the west Qinghai-Tibetan Plateau and Mediterranean regions (Xiang et al. 2021). East Asian *Eranthis* species (i.e., *E.byunsanensis*, *E.lobulata*, *E.pinnatifida*, and *E.stellata*) have *x* = 8. According to a recent phylogenetic study (Xiang et al. 2021), *E.sibirica* and *E.tanhoensis* are a derived group within the North Asian clade of *Eranthis* with non-canonical basic chromosome number *x* = 7 for the tribe Cimicifugeae.

The karyotypes of the two related species *E.stellata* and *E.tanhoensis*, with 2*n* = 16 and 2*n* = 14 chromosomes, respectively, are similar concerning five metacentric (I–V) and two submetacentric (VI–VII) chromosome pairs and differ by the presence of acrocentric pair (VIII) in *E.stellata*. It is well known that the basic chromosome number can change (dysploidy) due to chromosome rearrangements, fusion or fission of some chromosomes of the set and chromosome loss (Shubert 2007; Guerra 2008; Escudero et al. 2014). Dysploidy can establish powerful crossing barriers between sympatric taxa, as it disturbs regular chromosome pairing and bivalent formation at meiosis, drastically reducing hybrid fertility. These processes can result in the formation of new species (Grant 1981; Levin 2002; Baltisberger and Hörandl 2016). Such restructuring is known, for example, in the evolution of *Arabidopsisthaliana* (2*n* = 10) from *A.lyrata* (2*n* = 16) (Koch and Kiefer 2005). A similar case of descendant dysploidy was revealed for other Brassicaceae (Lysak et al. 2006) and plants from other families (Levin 2002). For Ranunculaceae, a decrease in the basic chromosome number from *x* = 8 to *x* = 7, caused by chromosome rearrangements, is known within *Ranunculus* (Baltisberger and Hörandl 2016) and *Anemone* (Mlinarec et al. 2012).

The shift to *x* = 7 in *Eranthis* possibly led to reproductive isolation of the populations with a new cytotype and, ultimately, speciation. We assume that further isolation of *E.tanhoensis* and *E.sibirica* was associated with polyploidization of the latter species. However, the type of polyploidy (i.e., autopolyploidy or allopolyploidy) has to be determined for this species. The karyotype of *E.sibirica* is similar to that of *E.tanhoensis* in chromosome morphology (metacentric and submetacentric chromosomes only), and they differ from the karyotypes of other related species. The organization of *E.sibirica* karyotype with 2*n* = 42 seems functionally diploid. The chromosomes are grouped in pairs (Fig. 3) and not in groups of 6. It is known that the size and shape of homologous chromosomes may change in the course of the diploidization process following polyploidization, i.e., due to the genome downsizing. Repetitive DNA sequences, both non-coding and coding, gene duplicates may be eliminated from the genome, resulting in changes in the karyotype parameters (Leitch and Bennett 2004; Mandáková and Lysak 2018; Wang et al. 2021).

A distinguishing feature of *E.tanhoensis* is the presence of small Bs in some of its populations (pops 11 and 14). Sporadic Bs were previously detected in individual cells only in *E.lobulata* (Erst et al. 2019). In some representatives of *E.tanhoensis*, up to 8 Bs could be observed in many cells. Bs are often found in representatives of Ranunculaceae and other families (Rice et al. 2015). However, their origin and possible adaptive and/or evolutionary roles are still poorly understood (Datta et al. 2016; Dhar et al. 2019). It is generally accepted that Bs are formed from A chromosomes in different ways. The most convincing case was the fully documented origin of a nascent B in trisomic *Plantagolagopus* L. from a supernumerary. This origin was associated with chromosome fragmentation, specific DNA sequence amplification, the addition of telomeric repeats and centromeric misdivision (Dhar et al. 2002). Bs could also escape as small centric fragments following unequal translocation and a reduction in chromosome number (Jones et al. 2008). Bs in *E.tanhoensis* may be preserved fragments of the lost ancestral pair VIII. The presence of Bs in the genome increases the adaptive capabilities of the population to adverse environmental conditions (Datta et al. 2016), which can be quite relevant for plants growing in this climatic zone.

### ﻿Genome size of *Eranthis*

The Kew list of DNA C-values contains only one C-value for white-flowered *Eranthis* (i.e., 1C = 8.20 pg) determined by Zonneveld et al. (2005) for *E.pinnatifida*. In the present study, we determined the genome size for six white-flowered *Eranthis* species. According to our data, the Japanese *E.pinnatifida* has an average 1C*x* = 9.80 ± 0.33 pg. It is the lowest absolute nuclear DNA content among the studied diploids. A lower 1C*x* value (9.26 ± 0.25 pg) was found only in the polyploid *E.sibirica*. Remarkably, a closely related diploid species, such as *E.tanhoensis*, shows 1C*x* = 12.48 ± 0.25 pg. According to the genome downsizing theory, an increase in the ploidy level leads to a decrease in the size of the monoploid genome. The loss of DNA in polyploids is a widespread phenomenon occurring in many plant groups (Shaked et al. 2001; Leitch and Bennett 2004; Adams and Wendel 2005). In the present study, *Eranthisstellata* exhibited the highest 1C*x*-value of 15.93 ± 0.32 pg and the highest total haploid length as well. However, it is interesting to note that, in this system, we found no significant correlation between 1C*x* and THL, as otherwise commonly found in plants (Levin 2002; Peruzzi et al. 2009), where this correlation typically exceeds r = 0.8. This inconsistency could be explained by different condensation degrees of the studied chromosomes. Nonetheless, it also may suggest differences in chromosomes width and volume (Kramer et al. 2021), not addressed in this study.

## ﻿Conclusions

In this study, the comparative karyomorphological analyses and genome size determination of six white-flowered species of Eranthissect.Shibateranthis from different populations have been carried out. The chromosome complements of *E.lobulata* and *E.byunsanensis* were determined for the first time. Karyotypes of studied *Eranthis* are shown to have both common features and species-specific features related to chromosome number, size and morphology. All the studied species can be distinguished based on their karyotype structure. They have the basic chromosome numbers *x* = 8 and *x* = 7, diploid and polyploid cytotypes. Additionally, *E.tanhoensis* and *E.lobulata* have small supernumerary chromosomes in the root tip cells. The monoploid genome size (C-value) determined by flow cytometry varies more than 1.5 times in the studied species.

## Supplementary Material

XML Treatment for Eranthis lobulata

XML Treatment for Eranthis stellata

XML Treatment for Eranthis tanhoensis

XML Treatment for Eranthis sibirica

XML Treatment for Eranthis byunsanensis

XML Treatment for Eranthis pinnatifida
